# Evaluation and modeling of diaphragm displacement using ultrasound imaging for wearable respiratory assistive robot

**DOI:** 10.3389/fbioe.2024.1436702

**Published:** 2024-08-16

**Authors:** Yan Zhang, Danye Li, Fengyao Zhang, Zongyu Wang, Lei Xue, Xiaolu Nan, Nianming Li, Xilai Tan, Weidong Guo, Yuru Zhang, Hongmei Zhao, Qinggang Ge, Dangxiao Wang

**Affiliations:** ^1^ The State Key Laboratory of Virtual Reality Technology and Systems, Beihang University, Beijing, China; ^2^ China-Japan Friendship Hospital, Beijing, China; ^3^ The Peking University Third Hospital, Beijing, China; ^4^ School of Mechanical Engineering and Automation, Beijing, China; ^5^ The Beijing Advanced Innovation Center for Biomedical Engineering, Beijing, China

**Keywords:** soft robot application, assistive robot, respiratory assistance, diaphragm ultrasonography, soft exoskeleton

## Abstract

**Introduction:**

Assessing the influence of respiratory assistive devices on the diaphragm mobility is essential for advancing patient care and improving treatment outcomes. Existing respiratory assistive robots have not yet effectively assessed their impact on diaphragm mobility. In this study, we introduce for the first time a non-invasive, real-time clinically feasible ultrasound method to evaluate the impact of soft wearable robots on diaphragm displacement.

**Methods:**

We measured and compared diaphragm displacement and lung volume in eight participants during both spontaneous and robotic-assisted respiration. Building on these measurements, we proposed a human-robot coupled two-compartment respiratory mechanics model that elucidates the underlying mechanism by which our extracorporeal wearable robots augments respiration. Specifically, the soft robot applies external compression to the abdominal wall muscles, inducing their inward movement, which consequently pushes the diaphragm upward and enhances respiratory function. Finally, we investigated the level and shape of various robotic assistive forces on diaphragm motion.

**Results:**

This robotic intervention leads to a significant increase in average diaphragm displacement by 1.95 times and in lung volume by 2.14 times compared to spontaneous respiration. Furthermore, the accuracy of the proposed respiratory mechanics model is confirmed by the experimental results, with less than 7% error in measurements of both diaphragm displacement and lung volume. Finally, the magnitude of robotic assistive forces positively correlates with diaphragm movement, while the shape of the forces shows no significant relationship with diaphragm activity.

**Conclusion:**

Our experimental findings validate the effective assistance mechanism of the proposed robot, which enhances diaphragm mobility and assists in ventilation through extracorporeal robotic intervention. This robotic system can assist with ventilation while increasing diaphragm mobility, potentially resolving the issue of diaphragm atrophy. Additionally, this work paves the way for improved robotic designs and personalized assistance, tailored to the dynamics of the diaphragm in respiratory rehabilitation.

## 1 Introduction

The diaphragm plays a pivotal role in respiration, accounting for up to 70% of inspiratory tidal volume in healthy individuals (Groth and Andrade, 2010; Howard, 2016). Severe diaphragm dysfunction or paralysis can lead to respiratory failure (Levine et al., 2008; Zorowitz, 2016). Recently, soft robotic actuators have been utilized as artificial muscles, capable of performing motions such as linear extension, contraction, bending, and twisting (Ali et al., 2021; Wen et al., 2024). These systems exert mechanical force or torque to either mimic or restore muscle movements, such as those of the diaphragm. Given their ability to replicate muscle movements, soft robotics is particularly promising for aiding in respiratory failure, offering potential improvements in mechanical ventilation devices.

Due to the diaphragm’s complex anatomical structure, enclosed by the ribcage and abdomen, previous studies have primarily utilized implanted soft robotic actuators that directly interact with the diaphragm to replace or augment its movement. Bashkin et al. (2007), Bashkin et al. (2009) report that a circle-shaped dielectric elastomer sheet, designed to completely replace the native diaphragm, bends downward and functions effectively as a pump diaphragm. Hu et al. (2023) report that a contractile soft robotic actuator, implanted above the diaphragm, augments its motion during inspiration while leaving the native diaphragm intact. These innovative solutions demonstrate the potential of mechanically supporting and augmenting diaphragm function using soft assistive robotics. However, despite these technological advances, the implantable nature of these devices introduces significant safety challenges.

Respiratory physiology demonstrates that applying pressure to the abdominal cavity can assist respiration (Konno and Mead, 1967). Inspired by this principle, wearable robotics designed to mimic human abdominal muscles offer a noninvasive method to improve breathing. For example, Lee et al. report on Exo-Abs (Lee et al., 2022), a belt-driven wearable robotic system that assists various respiratory functions by applying compensatory force to the abdominal muscles. In our previous work (Zhang et al., 2022), we employed an origami array to exert pressure on the human abdomen, achieving up to 12 cm of compression to further assist respiration.

Existing research has demonstrated initial success with soft robotics in respiratory assistance. However, most studies have primarily concentrated on the materials and fundamental functional design of these robots. There remains a relative scarcity of research on how these robots can precisely control diaphragm movement. Given the critical role of the diaphragm in respiration, it is crucial to develop a robotic-compatible, noninvasive, and real-time diaphragm monitoring system for ventilation support devices. Establishing such monitoring capabilities will provide valuable insights into the effectiveness and optimization of respiratory assistance provided by soft robotics.

To address such challenges in diaphragm monitoring in robotic ventilation, this article presents the first in-human use of diaphragm ultrasonography to quantitatively assess diaphragm displacement under noninvasive wearable robotic assistance. We record and characterize the deep tissue diaphragm displacement responses under this robotic assistance. Additionally, the human-robot coupled mechanical model provides a mechanism to assess changes in diaphragm displacement induced by extracorporeal robotic forces, and the precision of this model aligns with experimental results. As a result, the integration of ultrasound imaging technology and mechanical modeling effectively decodes diaphragm displacement under noninvasive robotic forces. The main contributions of this work are the following:

(1) Real-time, non-invasive, and precise assessment of the impact of respiratory assist devices on the diaphragm is crucial for ensuring the effectiveness and safety of these devices. Traditional methods like transdiaphragmatic pressure testing are often expensive and invasive (Raux et al., 2016), and electromyography of the diaphragm lacks intuitiveness (Zhu et al., 2015). Recently, functional ultrasound imaging has shown potential in monitoring diaphragm dynamics in complex environments due to its ease of use, widespread availability, non-invasive nature, and ability to detect deep tissues with high spatial resolution (Matamis et al., 2013; Gorman et al., 2002; Koco et al., 2021). Therefore, we utilize diaphragm ultrasound to measure and assess the effectiveness of wearable robotic interventions on diaphragm motion. The robotic diaphragm ultrasound evaluation facilitates a comprehensive understanding of the mechanisms behind extracorporeal robotic respiratory interventions. Specifically, the robot enhance respiration by externally influencing the abdominal muscles to assist diaphragm movement. Additionally, the robotic diaphragm ultrasound assessment provides crucial experimental data for subsequent modeling.
(2) Further, we propose a human-robot coupled two-compartment respiratory mechanics model to describe the pressure transmission process between the diaphragm-thoracoabdominal-lung system. This model is critical for quantifying how robotic interventions enhance diaphragm movement and respiratory function. It lays the foundation for predicting how the intervention of a soft robot alters this movement, thereby providing quantitative references for the control of the robot.
(3) Finally, looking toward future clinical translation, the patient may require varying degrees of assistance, such as different levels and patterns of pressurization, depending on the clinical condition. Thus, to further characterize the device, different pressurization shapes and levels were investigated, and the resulting diaphragm behavior was measured.

Based on our experiment findings, we believe this work will formulate a crucial step toward developing personalized, adaptive, diaphragm-synchronized respiratory assistance strategies and is expected to improve the adaptability, comfort, and effectiveness of respiratory devices for patients with respiratory diseases.

## 2 Diaphragm ultrasound evaluation for soft respiratory robot

### 2.1 Evaluation of diaphragm displacement using ultrasound imaging for soft respiratory robot

**FIGURE 1:**
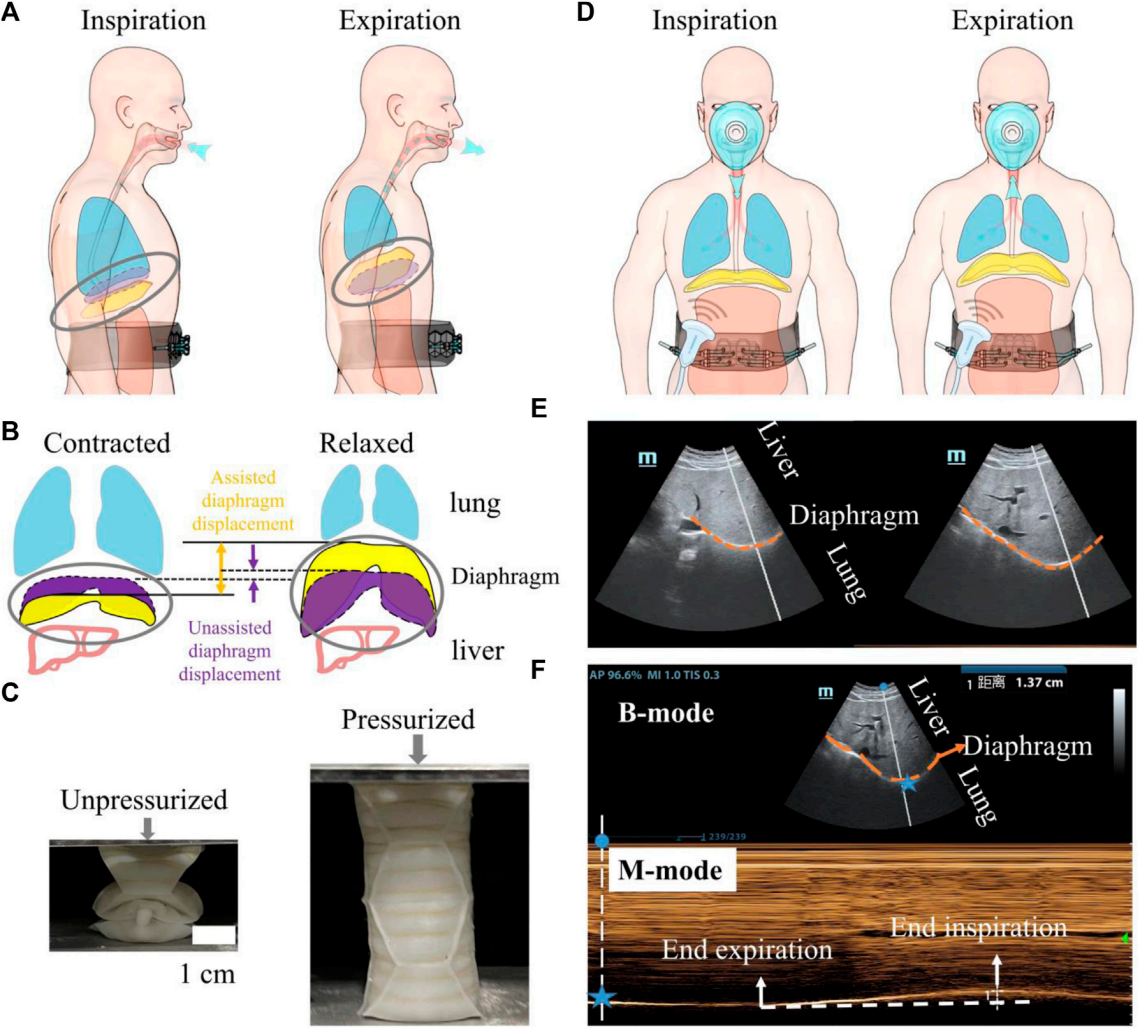
An overview of the utilization of a wearable robot to augment diaphragm motion, with an evaluation conducted through diaphragm ultrasound during robotic-assisted ventilation. **(A)** A soft wearable robot augments diaphragm motion by applying pressure to the abdominal muscles. **(B)** Schematic depicting diaphragm motion during respiration. The dotted purple volume represents the diaphragm’s contracted and relaxed states during natural breathing. The solid yellow area indicates the movement of the diaphragm assisted by the robot. The distance indicated by the arrows represents the movement of the diaphragm during natural breathing and under robotic assistance. **(C)** A single origami actuator in a unpressurized for fitting the human body (left) and in a pressurized state for augmenting diaphragm motion (right). The soft respiratory robot is composed of eight origami actuators and origami arrays are constrained within the non-stretchable strap to expand inward when actuated. **(D)** Probe position for diaphragmatic excursion measurements. **(E)** B-mode diaphragm sonography. The bright curve highlighted with orange dash line reflects the diaphragm; diaphragm moves toward the probe during inspiration. **(F)** M-mode diaphragm sonography. The peaks and valleys indicate the beginning and the end of the diaphragmatic contraction. The amplitude indicates an excursion (displacement) of 1.37 cm. The horizontal axis represents inspiratory time. For all images, the probe was positioned in the right subcostal space, pointing toward the cranial direction. Blue star represents the spatial location of the diaphragm.

As depicted in Figure 1A, the diaphragm plays a crucial role in the respiratory process. When the diaphragm contracts, the diaphragm’s arclength reduces, leading to a downward movement of the diaphragm sheet. This movement increases the volume of the thoracic cavity and decreases pressure, facilitating inhalation. On the contrary, during forced expiration, the diaphragm relaxes and moves upward, while the abdominal muscles contract inward. This action reduces the volume of the abdominal cavity, leading to air exhalation (Figure 1B).

In our previous work (Zhang Y. et al., 2022), we harness the deployable function of pneumatic soft origami actuators to enhance abdominal muscle retraction. The increase of pressure difference between abdominal and thoracic cavities will improve exhalation and augment the native movement of the diaphragm (Figure 1B). Inhalation is performed by the pressure difference between the atmosphere and the lung when the soft origamis transform to unpressurized state. With the assistance of robot, the range of diaphragm motion during respiration will be significantly expanded.

As depicted in Figure 1C, we opt for the Yoshimura origami cylinder actuator because of its strengths in symmetric growth in the axial direction. The soft actuators, under 40 kPa pressurization, are capable of generating an output force of up to 200 N. This force is sufficient to compress the abdomen inward by 6–12 cm.

We use diaphragm ultrasound in robotic ventilation to validate: 1) whether the soft robot can generate sufficient forces that overcome skin-musculoskeletal resistance and augment diaphragm displacement; 2) whether the displaced diaphragmatic volume can increase inspiratory driving forces and improve ventilation.

Ultrasonography is utilized to visualize and measure the motion of the diaphragm under robotic assistance [(Figure 1D)]. Two-dimensional (B-mode, brightness) ultrasonography (Figure 1E) is employed to display the diaphragm’s motion. To precisely quantify the diaphragm’s displacement, M-mode ultrasonography is used. This method visualizes movement along a selected line within the B-mode image over time, providing a detailed view of the diaphragm’s dynamic responses to the actuation by the soft robot (Figure 1F).

### 2.2 Respiratory mechanic modelling for diaphragm displacement estimation

Unlike direct interventions on the diaphragm, the proposed soft robot facilitates diaphragmatic movement through pressure transmission, altering the pressure distribution within the thoracoabdominal cavity. This mechanism boosts the effective movement of the diaphragm, potentially improving pulmonary function. To deeply understand and validate this process, we developed a human-robot coupled two-compartment respiratory mechanics model to estimate diaphragm displacement and lung ventilation. This modeling approach not only scientifically assesses the impact of robotic assistance on diaphragmatic activity but also provides a crucial scientific foundation for evaluating and optimizing robotic-assisted respiratory therapy.

**FIGURE 2:**
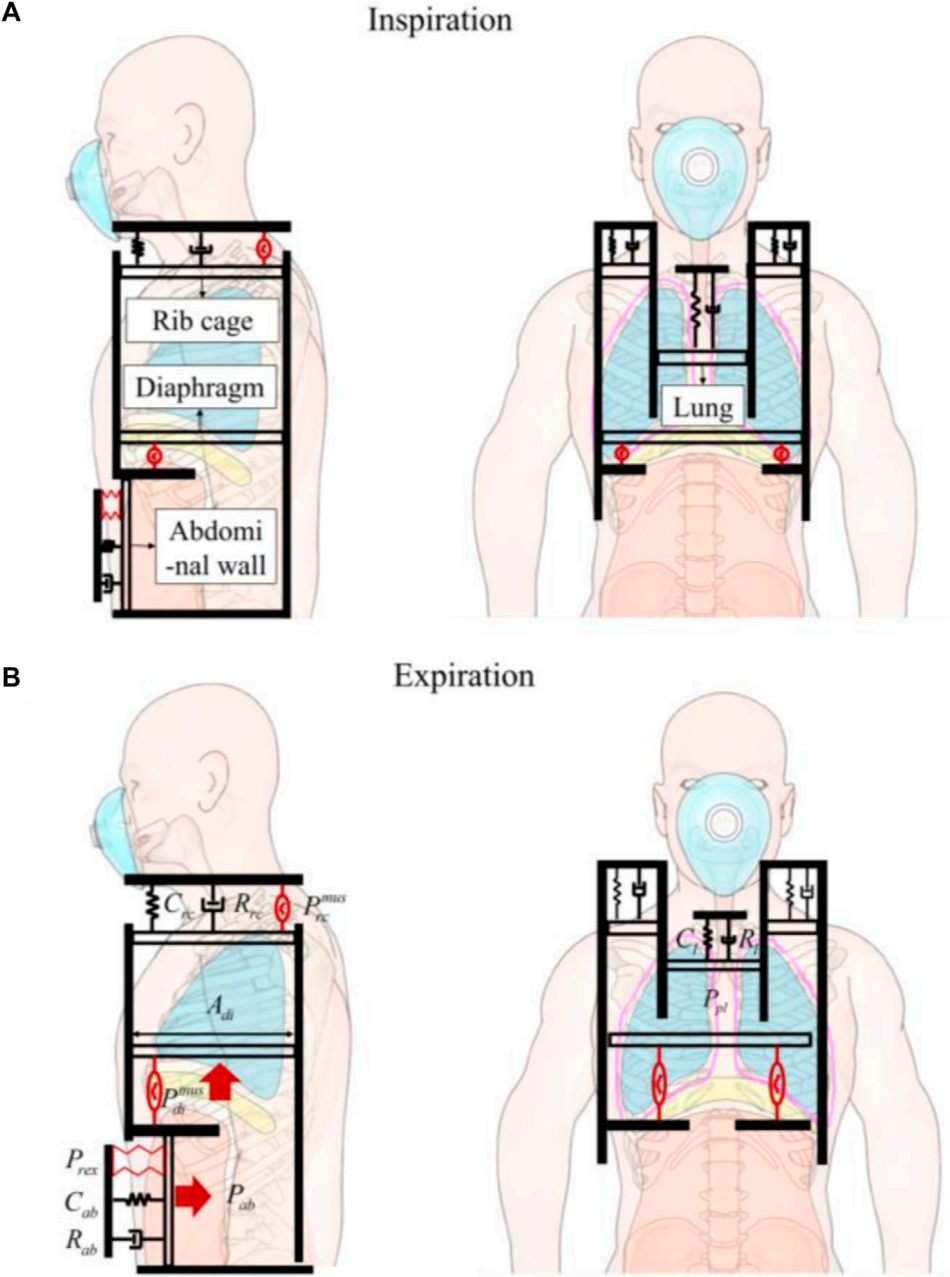
The human-robot coupled two-compartment respiratory mechanic model. **(A)** Inspiration. **(B)** Expiration. Four pistons represent rib cage, abdominal wall, diaphragm, and the lung. The pistons are anchored to a stationary foundation, represented by a solid black wall, and experience displacements due to the forces exerted on them. The driving pressure exerted by the rib cage muscles ( $P_{rc}^{mus}$ ) displaces the rib cage piston upward, and the force exerted by the diaphragm muscle ( $P_{di}^{mus}$ ) pulls the diaphragm piston downward, while the robotic forces (*P*_*rex*_) push the abdomen piston inward. The pleural cavity between the lung and chest wall is outlined in pink and its pressure denoted as (*P*_*pl*_). The pressure difference between the pleural cavity and atmospheric pressure drives the movement of the lung piston. The damper and spring represent the resistances and the elasticity of the compartments.

Based on the respiratory mechanics principles described by Konno and Mead (1967), who proposed the concept of the chest wall as a two-compartment system, our model integrates these concepts into the modern context of robotic assistance. This two-compartment model includes the rib cage and abdomen, separated by the diaphragm. The pleural cavity, a compliant small-volume structure, connects the lung surfaces with the rib cage and diaphragm. The movements of the ribs and diaphragm cause changes in the pressure within the pleural cavity, which in turn transmits these movements to the lungs, facilitating the process of respiration (Figure 2A).

We assume that the contents of the abdomen are incompressible, bounded dorsally by the spine, caudally by the pelvis, and laterally by the iliac crests. Essentially, only the ventral abdominal wall and diaphragm can be displaced. When the diaphragm descends, abdominal pressure (*P*_*ab*_) increases, pushing the ventral abdominal wall outward. Conversely, when the abdominal muscles contract, the ventral abdominal wall moves inward, increasing *P*_*ab*_ and causing the diaphragm to move cranially (Figure 2B).

Therefore, our model aims to intervene in the inward movement of the abdominal wall through robotic technology. This intervention, by causing displacement of the diaphragm, leads to changes in the pressure within the pleural cavity, thus effectively intervening in respiration. Further, we present an approach for human-robot coupled respiratory modeling by taking into account the specific robotic components involved.

The dynamics associated with the expansion of rib cage cavity, driven by the inspiratory muscle ( $P_{rc}^{mus}$ ), can be written as:

$$R_{rc}{\dot{V}}_{rc}+\frac{V_{rc}}{C_{rc}}=P_{rc}^{mus}$$ (1)

Where *V*_*rc*_ denotes the increase in rib cage volume driven by the inspiratory muscles ( $P_{rc}^{mus}$ ), which accommodates the expanding lungs during inspiration. *C*_*rc*_ and *R*_*rc*_ represent the compliance and resistance of rib cage.

For the abdomen cavity, the robot forces of expiratory module (*P*_*rex*_) collaborate with diaphragm forces ( $P_{di}^{mus}$ ) to increase abdominal pressure,

$$P_{di}^{mus}+P_{rex}=P_{ab}$$ (2)

The dynamics associated with abdominal cavity can be represented as:

$$R_{ab}{\dot{V}}_{ab}+\frac{V_{ab}}{C_{ab}}=P_{ab}$$ (3)

Here, *V*_*ab*_ represents the changes in abdominal volume occurring in the thoracoabdominal junction, which can affect lung function. These changes can result from both diaphragm muscle activity and the robotic forces applied to the abdominal wall. Although the total volume of the abdominal cavity remains unchanged, *V*_*ab*_ specifically denotes the changes in diaphragmatic piston. *C*_*ab*_ and *R*_*ab*_ represent the compliance and resistance of abdomen.

Since the expanding or contracting chest and the descending and relaxing diaphragm create space that alters the pressure within the pleural cavity, we have

$$\frac{V_{ab}+V_{rc}}{C_{pl}}=P_{pl}$$ (4)

*C*_*pl*_ and *P*_*pl*_ represent the compliance and pressure of pleural cavity.

The lung dynamics can be written as

$$R_{l}{\dot{V}}_{l}+\frac{V_{l}}{C_{l}}=P_{pl}$$ (5)

Here, *C*_*l*_ and *R*_*l*_ represent the compliance and resistance of abdomen, rib cage the lung.

Rewriting Equations 1–5 in matrix form, we obtain Equation 6.

$$\left\{\begin{array}{l} {\dot{V}}_{rc}=\frac{1}{R_{rc}}\left(-\frac{1}{C_{rc}}V_{rc}+P_{rc}^{mus}\right)\\ {\dot{V}}_{ab}=\frac{1}{R_{ab}}\left(-\frac{1}{C_{ab}}V_{ab}+P_{di}^{mus}+P_{rex}\right)\\ {\dot{V}}_{l}=\frac{1}{R_{l}}\left(-\frac{1}{C_{l}}V_{l}+\frac{V_{ab}+V_{rc}}{C_{pl}}\right) \end{array}\right.$$ (6)

The inward motion of the ventral wall of the abdomen will produce equivalent displaced diaphragmatic volume and the cranial displacement of the diaphragm can be calculated according to Equation 7.

$$x_{di}=\frac{V_{ab}}{A_{di}}$$ (7)

$A_{di}$ represents the cross-sectional area (square meters) of human body which approximates individual’s weight divided by the height.

## 3 Materials and methods

### 3.1 Design and fabrication of soft origami actuators

In contrast to our previous work, where soft robots were employed for expectoration assistance requiring them to deliver impacts of over 200 N within 200 milliseconds to rapidly increase pulmonary pressure and peak expired flow, the use of robots for respiratory assistance demands higher mechanical performance. Specifically, because breathing is a continuous and real-time process, it is essential to significantly enhance the robot’s fatigue resistance capabilities when they are adapted for this new application.

**FIGURE 3:**
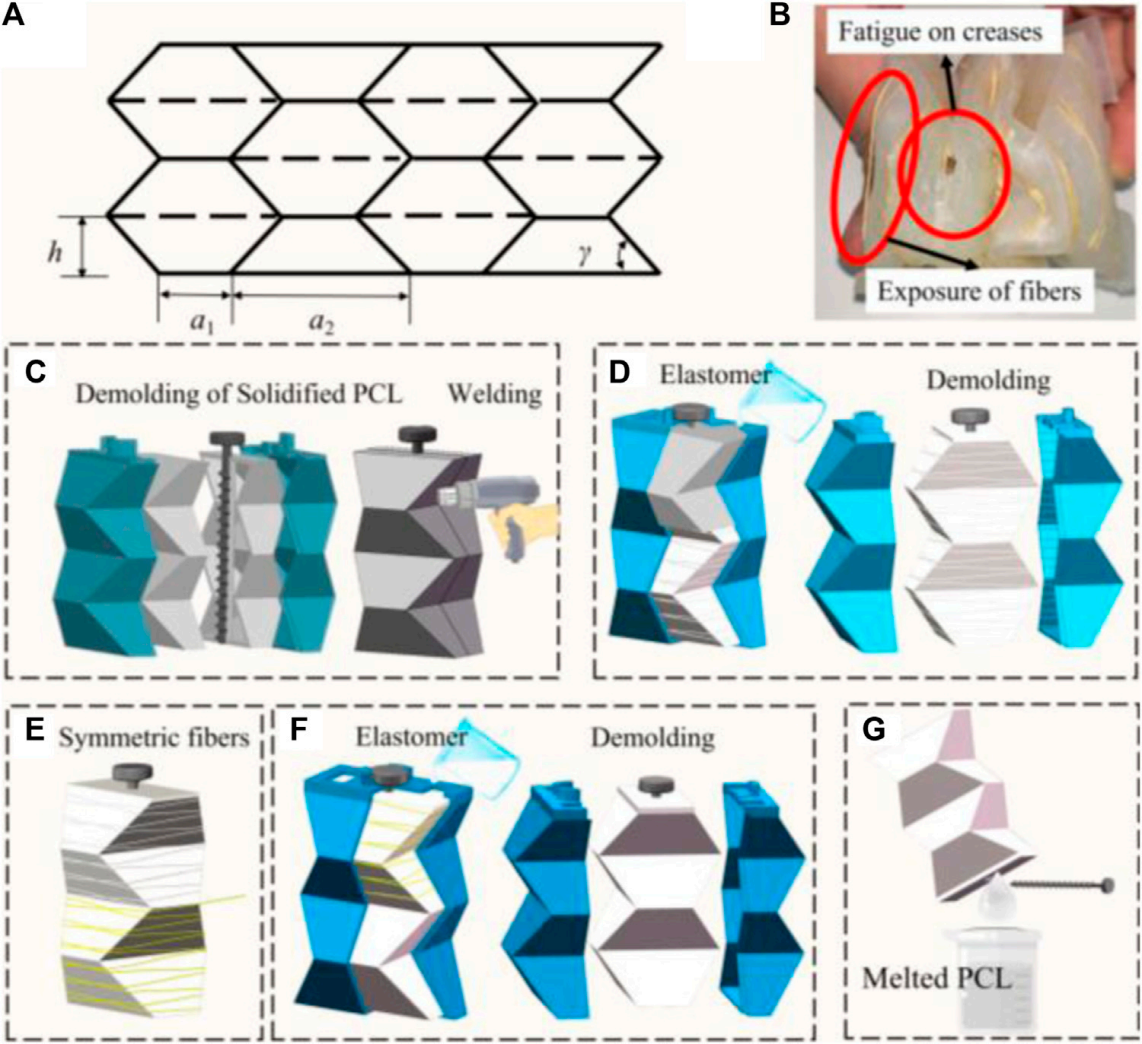
**(A)** Crease pattern of Yoshimura origami. **(B)** Surface defects of soft actuator. **(C–G)** Optimized process of fiber-reinforced, double-layered integrated fabrication method with PCL.

For the Yoshimura origami structure, the main parameters are shown in Figure 3A, including the parallel sides of the equilateral trapezoid *a*_1_ and *a*_2_, and hight of the trapezoid *h*, and based angle *γ* (Seo et al., 2021; Zhang et al., 2022; Zhang et al., 2023). For a closed tube, four trapezoids form a basic foldable element. The pattern consists of *N* layers and the number of horizontal trapezoids is *M*. *θ* is the pre-folding angle of the pattern. The closure condition of Yoshimura origami can be written as Equation 8.

$$\tan\gamma \cos\frac{\theta }{2}=\tan\frac{\pi }{M}$$ (8)

In our previous work (Zhang et al., 2022), we identified that the geometric parameters of the Yoshimura origami, with *a*_1_ set to 2 cm, *γ* at 60°, and both *M* and *N* equal to 4, facilitate easy compression and expansion in the vertical direction simultaneously.

An actuator’s fatigue life critically influences its applicability. To evaluate the actuator’s endurance, we subjected it to a fatigue test involving a square wave pressure input, alternating between 3 s of inflation and 3 s of deflation. It was observed that the actuator fails to sustain pressures exceeding 20 kPa beyond 20 cycles, with predominant failure points located at the creases due to stress concentration, as illustrated in Figure 3B. To enhance its viability for respiratory support applications, we increased the thickness of the actuator’s outer silicone rubber layer $\delta_{o}$ .

Monolithic formation technology enhances pressure capacity of actuator and prevents surface defects. Previously, the lost-wax process was utilized to create hollow structures; however, the presence of wax particles has been found to compromise the surface quality of silicone, diminish the adhesion between silicone and other materials (such as gas pipes), and contribute to environmental pollution. Herein, polycaprolactone (PCL) is introduced as a substitute for wax (Guarino et al., 2002; Gul et al., 2018; Girao et al., 2018). PCL, a non-toxic polymer with a melting point of 60°C and a sufficiently high boiling point, transitions to a gelatinous state upon heating and solidifies at a rate conducive to forming.

The optimized process of fiber-reinforced, double-layered integrated fabrication method is depicted in Figure 3. It involves preparing 3D-printed molds for the inner PCL mold with a rod, an external mold, and an outer mold. Initially, PCL particles are melted using hot water above 80°C, and the molten PCL is shaped into two-halves of the mold (as shown in Figure 3C), which are then allowed to cure at room temperature for approximately 5 min. The halves are joined by melting and curing their edges with a hot air gun, creating a Yoshimura-origami PCL core integrated with a carbon fiber rod for precise alignment in subsequent steps. A rod with a screw thread is specifically selected to prevent unwanted rotation and enhance positioning accuracy. The solid PCL Yoshimura-origami core is then encased within the external molds, secured in place by the rod and a locating boss. Elastomer is poured into the outer mold and cured to embed the PCL core within the inner silicone layer (Figure 3D). As Figure 3E illustrates, fibers are manually attached to the silicone core using inextensible Kevlar thread in a symmetrical double helix configuration for added stability. The outer silicone skin is then formed using the outer mold, as demonstrated in Figure 3F. Following a similar curing process for the outer layer, the actuator is immersed in hot water above 80°C to liquefy the inner PCL core, allowing the carbon fiber rod to be removed once the PCL has fully melted. The molten PCL is then extracted from the silicone, as shown in Figure 3G.

To evaluate this enhancement, we produced three actuators for each predefined dimension. We verified the actuator’s performance through a fatigue test, defining failure as its inability to sustain a pressure of 30 kPa. This pressure threshold was chosen because the soft origami actuator consistently generated an effective assistive force of 200 N at 30 kPa, comparable to the manual abdominal thrust performed by a respiratory therapist. We then tracked the number of cycles until the actuators failed, with failure defined as the inability to sustain a pressure of 30 kPa. The results, presented in Table 1, reveal that actuators with a wall thickness of 2 mm meet our performance standards. Therefore, we have set a minimum wall thickness of 2 mm as a standard, achieving an optimal balance between durability and the necessity for lightweight design.

**TABLE 1:** The four sets of dimensions tests.

*Parameters*	$\delta_{o,1}=1\text{ mm}$	$\delta_{o,2}=1.5\text{ mm}$	$\delta_{o,3}=2\text{ mm}$	$\delta_{o,4}=3\text{ mm}$
*a*_1_/mm	51.95	53.17	54.40	56.85
*a*_2_/mm	23.67	24.89	26.12	28.57
Available cycles with 30 kPa	∼20	∼30	>100	>100
Surface after Test	Breaks on creases	Breaks and flaws	Few flaws, no breaks	Few flaws, no breaks

Finally, in order to avoid pressure concentrations that could lead to injuries to the body, our actuator consists of 8 soft actuators side by side, 4 in each row, covering the upper abdomen of the body. The soft origami arrays are mounted near the base line of the raphe muscles and do not interfere with the neighboring ribs.

### 3.2 Performance of pneumatic control system of soft robot

In a clinical setting, the degree of assistance provided by a robot for respiratory support should be adjustable based on the clinical situation and the patient’s condition. The level of assistance delivered by the device is primarily determined by the pressure settings of the soft origami structures, which is set by the pneumatic control system. This section thoroughly evaluates the ceiling limits of robot system and lays the groundwork for subsequent studies on the effects of various robotic parameters on diaphragm displacement.

The control system employs an Arduino Mega 2560 to interface with a host computer through a USB serial port for command reception. It manages two solenoid valves—a supply valve and an exhaust valve—for the origami actuators. Due to the rapid time response requirements, Metal-Oxide-Semiconductors (MOS) are utilized as control switches. Each MOS’s input end is connected to the digital output serial port of the Arduino, controlling the opening and closing of the valves by regulating the high and low voltage levels. The valves are powered by the power supply and connected in series to the output terminals of the MOS, each controlled by corresponding inputs.

Air pressure in the actuator modules is monitored by pressure sensors, which enable the Arduino to adjust gas flow by opening or closing valves as necessary. The system employs an XGZP6847A200KPG analog pressure sensor for monitoring positive pressure. These sensors are connected directly to the actuator modules via air pipes, sending real-time analog voltage signals to the Arduino.

The closed-loop control logic involves setting a target pressure and a threshold. For the soft origami actuators, if the air pressure falls below the target minus the threshold, the supply valve opens to inflate it. If the pressure exceeds the target plus the threshold, the exhaust valve opens to release air. If the pressure is within the threshold, both valves close to maintain the current pressure. The Arduino continuously adjusts the air pressure in the actuator modules in a closed-loop system, following predetermined waveforms and pressure settings. This ensures rapid attainment and maintenance of the desired air pressure.

**FIGURE 4:**
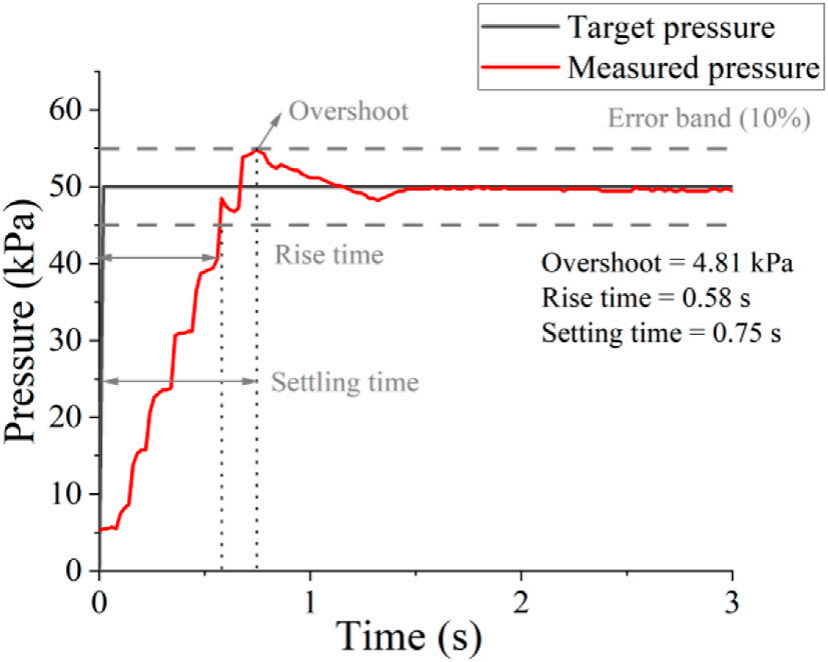
Low-level control performance without load. Control response to a maximum pressure step (0–50 kPa).

Figure 4 shows the response to a 50 kPa pressure step of the robotic control system, a worst case given that it represents the maximum pressure step possible for this study. The error band is calculated as the 10% of error of the desired pressure. Rise time is when measured pressure is first at 10% of the target time; settling time is when measured pressure is settled within 10% of the error of the target pressure (Proietti et al., 2023). Our pressure supply and controller exhibit minimal overshoot (less than 4.81 kPa, within the 10% error of the target) and rapid settling time (less than 0.75 s), which is ideal for assisting respiratory movements that typically ranged from 3 to 6s per breath cycle.

Actuator behavior is controlled by the degree of pressurization. Preset pressurization waveforms are programmed into the control system and electropneumatic regulators. The accuracy in replicating these idealized waveforms is constrained by the control resolution of the electropneumatic regulators, ultimately leading to the generation of output pressurization curves. These pressure curves for actuation ultimately dictate the mechanical performance of the actuators.

### 3.3 Ultrasound imaging of the diaphragm and its associated displacement with and without robotic assisted ventilation

This section introduces, for the first time, the evaluation of wearable robots for respiratory assistance using diaphragm ultrasonography. This method extends the assessment of respiratory assistance robots from airflow signals to the level of muscle dynamics.

**FIGURE 5:**
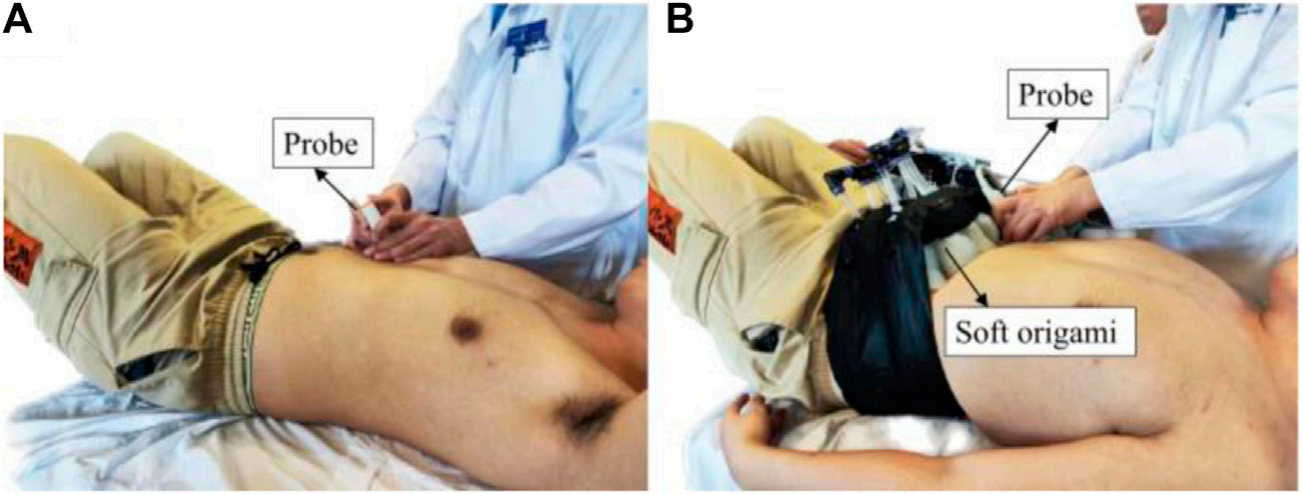
Experimental setup. **(A)** Spontaneous respiration and **(B)** robotic assistive respiration.

We use a 2–5 MHz phased convex array probe (Mindray M9, China). The probe is aimed at the top of the diaphragm, placed at the junction of the anterior axillary line or the midclavicular line and the costal margin (Figure 5). Two-dimensional B-mode is selected to locate diaphragm and the white dash line should be perpendicular to the diaphragm to achieve high-quality image. We use M-mode ultrasonography to quantify and visualize the motion of diaphragm over time due to its excellent axial and temporal resolutions.

Diaphragm displacement reveals information about the respiratory biomechanics that physically drives ventilation. All ultrasound measurements were conducted by an experienced sonographer. The right hemidiaphragm was selected due to the limited acoustic window on the left side. The ultrasound picture was captured while breathing steadily and three regular breath cycles were displayed; the sonographer selected one of the brightest curves and measured the diaphragm displacement using the imaging processing software accompanied by the diaphragm ultrasound equipment.

To evaluate the ability of extracorporeal robot assistance to augment respiratory function, the participants were asked to perform respiratory tasks; besides, respiratory physiological data including respiratory flow and diaphragm ultrasound were recorded simultaneously. Flow is accurately measured using a spirometer and tidal volume is derived by integrating flow over time. During the respiratory task, participants were asked to breathe spontaneously then conduct robotic assistive respiration. For healthy adults, the typical respiratory cycle range is 3∼6 s (Yang et al., 2022) and we set 3 s for robotic inflation during expiration and deflated the actuators during inhalation. To ensure synchronized robotic actuation with the subject’s natural respiratory rhythm, participants with no prior experience in robotic-assisted respiration underwent effective human-robot synchronization training, involving 15 breath cycles.

## 4 Results

### 4.1 Diaphragm ultrasound evaluation under soft robot assistance

**FIGURE 6:**
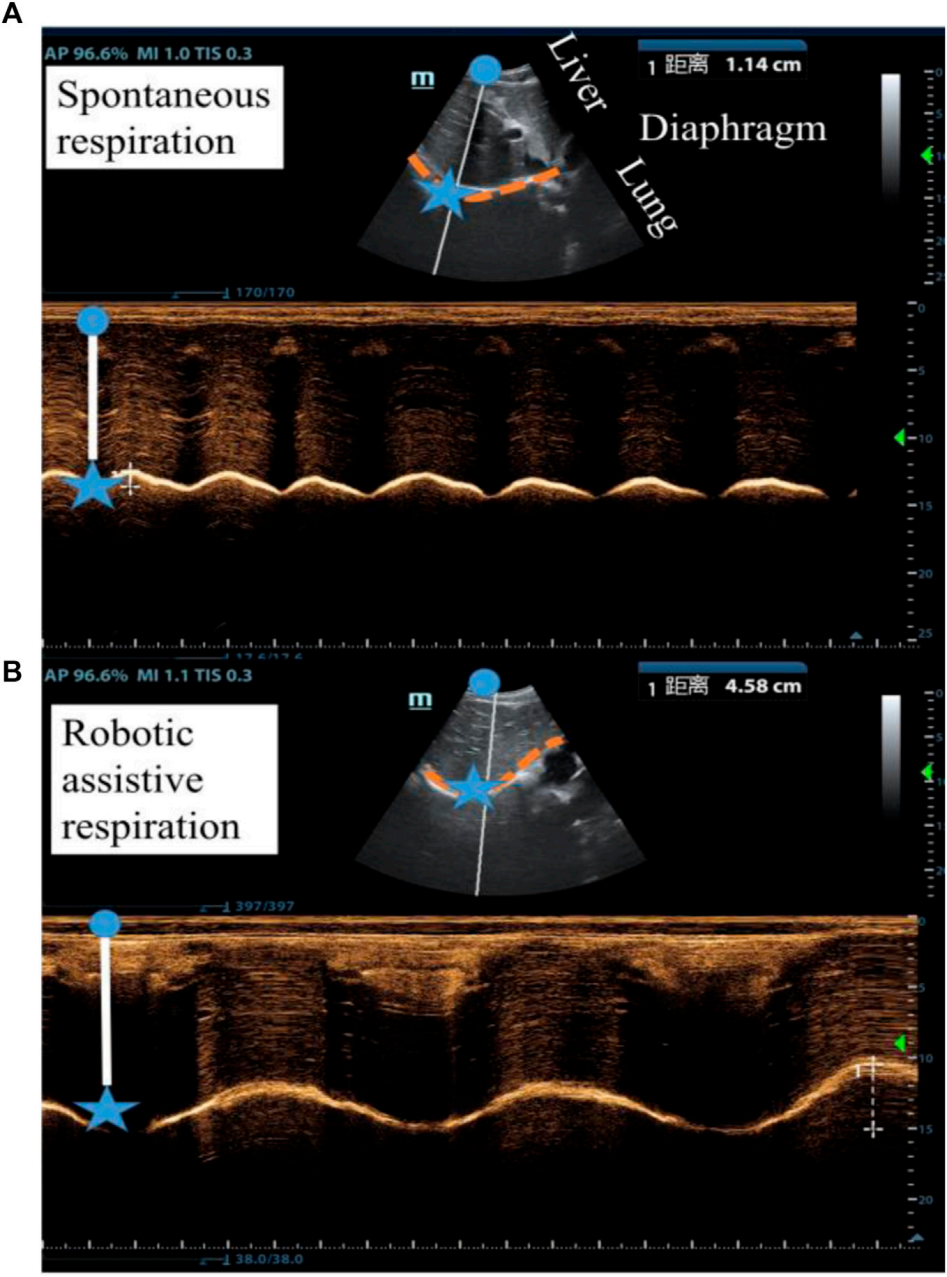
Ultrasound imaging of the diaphragm displacement **(A)** without robotic assistance and **(B)** with robotic assistance. (Participant 6).

**FIGURE 7:**
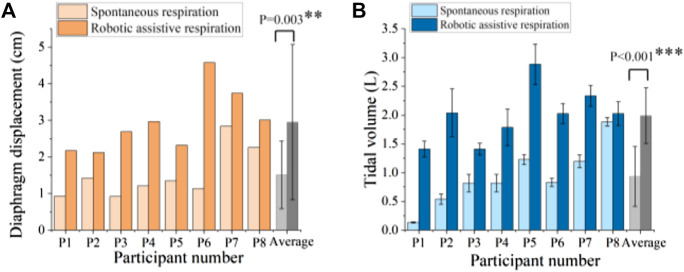
Robotic ability to augment **(A)** diaphragm displacement and **(B)** tidal volume. ** denotes *p* < 0.05, *** denotes *p* < 0.001.

We used ultrasound imaging to look “under the skin” and measure how robotic actuation alters diaphragm dynamics. We simultaneously collected data on diaphragm displacement using ultrasound and lung volume using a spirometer with and without robotic assistance. From the typical subject (Participant 6), the actuator augments the diaphragm displacement per breath from 1.14 cm displacement of unassisted ventilation to 4.58 cm displacement of assisted ventilation and decrease the breath rate (Figures 6A, B). The flow data shows that the soft robot has the direct capacity to augment tidal volume from 0.83 L to 2.03 L (Figure 7).

Figure 7 shows the comparison of spontaneous and robotic assistive breath in terms of diaphragm displacement and tidal volume for 8 participants. The results demonstrated that the soft robot can significantly increase 1.95 times diaphragm displacement and 2.14 times tidal volume on average compared with spontaneous breath.

**FIGURE 8:**
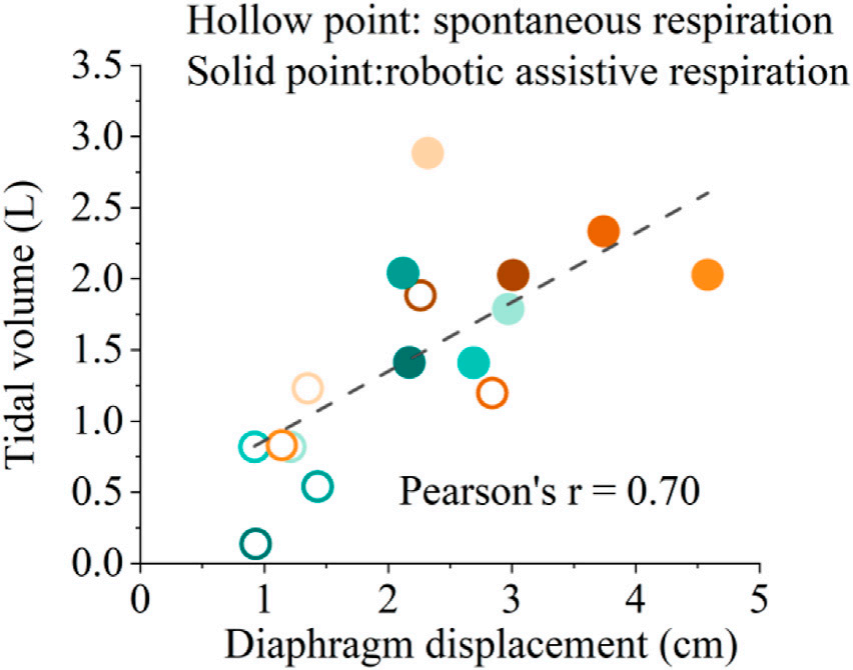
Correlation between diaphragmatic displacement and tidal volume during spontaneous respiration and robotic assistive respiration. Different colors represent the eight different healthy subjects.

Figure 8 shows the correlation between diaphragmatic displacement and tidal volume during quiet breathing and robotic assistive breath. The results illustrate that the diaphragm displacement significantly correlated with tidal volume for 8 subjects, validating the effectiveness of soft robotic ventilation through diaphragm ultrasound.

Figure 8 shows the solid points represent robotic assistive conditions are distributed in the upper right region of the diaphragm displacement-tidal volume correlation diagram. The results show that soft robot can simultaneously augment diaphragm displacement and enhance ventilation. This implies a synergistic interaction between the soft robotic device and the human diaphragm, ultimately resulting in an improved ventilation efficiency. Such an augmentation could be particularly pertinent for individuals necessitating respiratory support, such as those afflicted by specific respiratory disorders.

### 4.2 Validation of human-robot coupled two-compartment respiratory mechanic model

Building upon the diaphragm ultrasound measurements of soft robotic assistive respiration, we introduced a two-compartment mechanical model to quantitatively depict the extracorporeal robotic respiratory assistance mechanism. To evaluate the model’s accuracy, we compared the analytical and experimental results. The model provides insights into the dynamic behavior of the diaphragm-thoracoabdominal cavity-lung system during the robot’s respiratory assistance.

The muscle driving forces can be presented as a trigonometric function (Zhang et al., 2022); we use the robotic square driving pressure to produce synchronized compressive forces along with the abdominal muscles; simulation parameter values of respiratory mechanic model are listed in Table 2.

**TABLE 2:** Simulation parameter values of respiratory regulation.

*Parameter*	*Value*	*Parameter*	*Value*	*Parameter*	*Value*
*R*_*l*_ (cmH_2_O/(L·s))	2	*C*_*l*_ (L/cmH_2_O)	0.2	*C*_*pl*_ (L/cmH_2_O)	0.25
*R*_*ab*_ (cmH_2_O/(L·s))	1	*C*_*ab*_ (L/cmH_2_O)	0.4	*P*_*rex*_ (cmH_2_O)	3
*R*_*rc*_ (cmH_2_O/(L·s))	1	*C*_*rc*_ (L/cmH_2_O))	0.2	*A*_*di*_ (m^2^)	0.08

**FIGURE 9:**
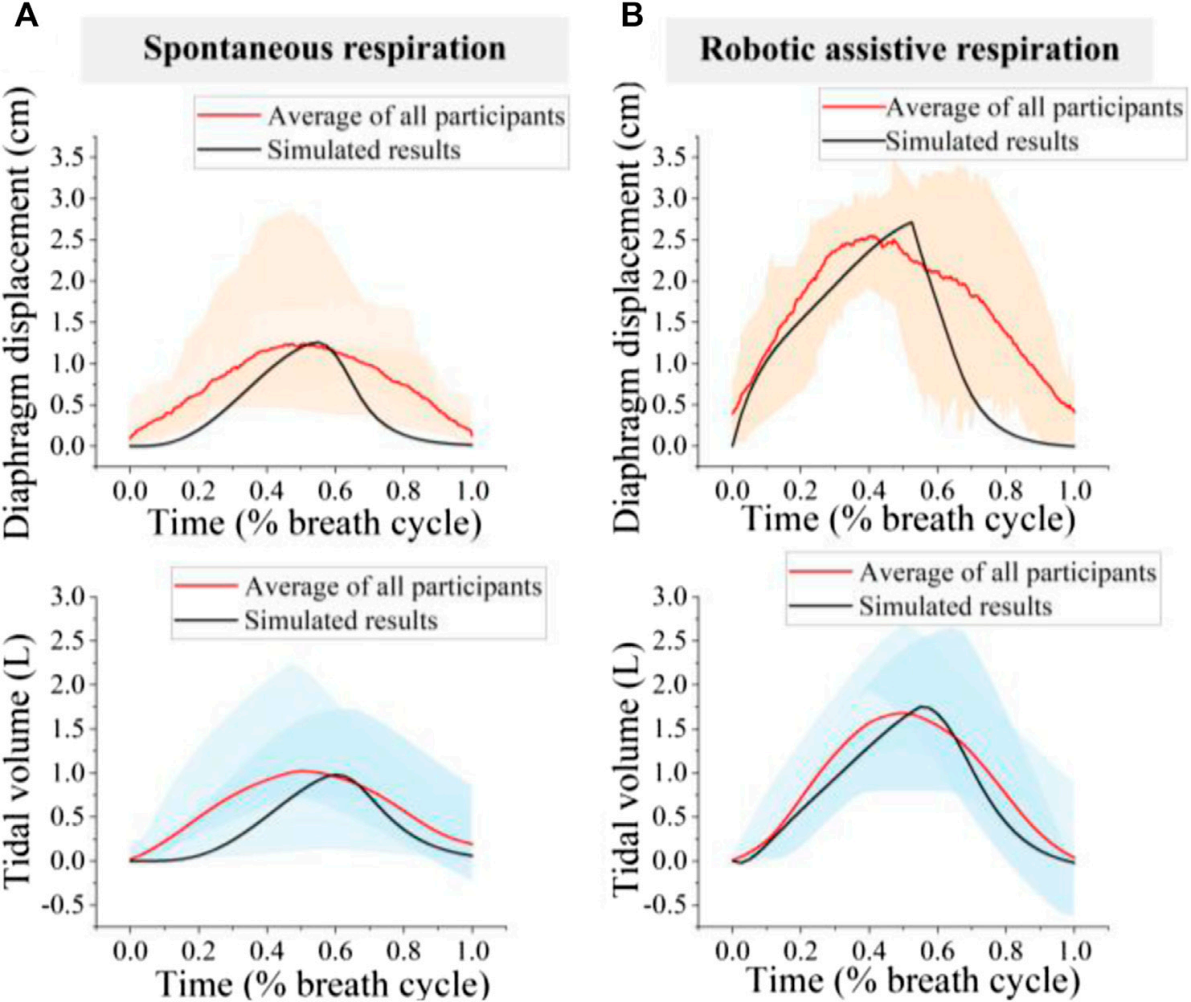
Comparison of simulated and experimental results **(A)** spontaneous respiration; **(B)** Robotic assistive respiration. The orange area represents the range of diaphragm displacement and the blue area represents tidal volume for 8 participants.

Average diaphragm displacement profiles from ultrasound and tidal volume profiles from spirometer over the entire pool of participants are shown in Figure 9. The amplitude error for diaphragm displacement and tidal volume estimation between simulation and experiment is less than 7%.

The patterns of diaphragm displacement curve and tidal volume waveform, both with and without robotic assistance, exhibited significant variation across participants (Figure 9). The normalized root means square errors (NRMSEs) less than 31.8% for diaphragm displacement estimation and 27.6% for tidal volume estimation. The variation in diaphragm motion patterns and tidal volume waveforms for spontaneous respiration among individuals may be attributed to physiological and neurological differences (Figure 9A). Therefore, the difference may also cause divergent responses to the same device, leading to the robotic assistive respiration patterns varied widely across participants (Figure 9B).

### 4.3 Effect of robotic pressure patterns on diaphragm displacement

In order to gain a comprehensive understanding of the impact of robotic-assisted ventilation systems on respiratory dynamics, it is imperative to further explore the effects of different pressurization patterns on diaphragm motion. By delving into these influences, we can enhance our understanding of the mechanics behind robotic-assisted ventilation systems and provide insights for designing more effective respiratory support devices. Therefore, this section will focus on elucidating the effects of different pressurization patterns on diaphragm displacement curves and the significance of these findings for respiratory support system design.

**FIGURE 10:**
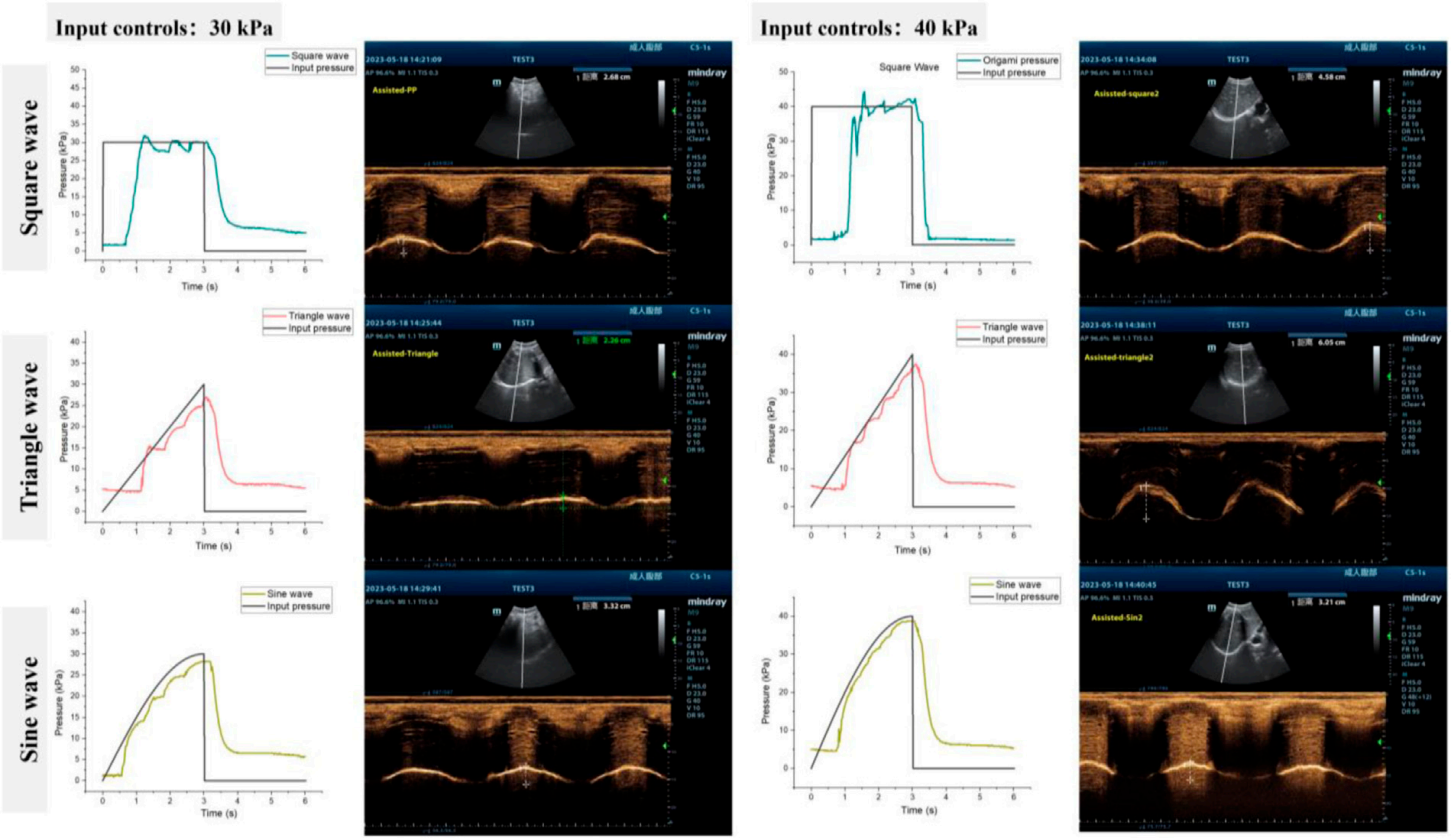
Tuning actuation pressurization for different pneumatic waveforms **(A)** 30 kPa and **(B)** 40 kPa.

As a proof-of-concept, we examined the response of a single subject (participant 6) to different pressurization patterns. As depicted in Figure 10, actuator characterization was performed both *in vitro* and *in vivo*. For the *in vitro* characterization, we evaluated the pressure response of soft origami actuators using the method described in Section 3.2. We measured the pressure response of soft origamis under three of the most commonly used pressurization waveforms in clinical mechanical ventilation: square, triangle, and sine waves, which serve as representatives. The mean absolute error (MAE) of the pneumatic system for square, triangle, and sine waves are 10.71, 9.36 and 6.78 kPa respectively.

The performance of the diaphragm-assist system was analyzed *in vivo* by assessing diaphragm movement through ultrasonography and evaluating lung capacity through metrics such as tidal volume. Different pressurization shapes and levels were programmed into the actuator, with the ensuing diaphragm ultrasound images being recorded as Figure 10.

**FIGURE 11:**
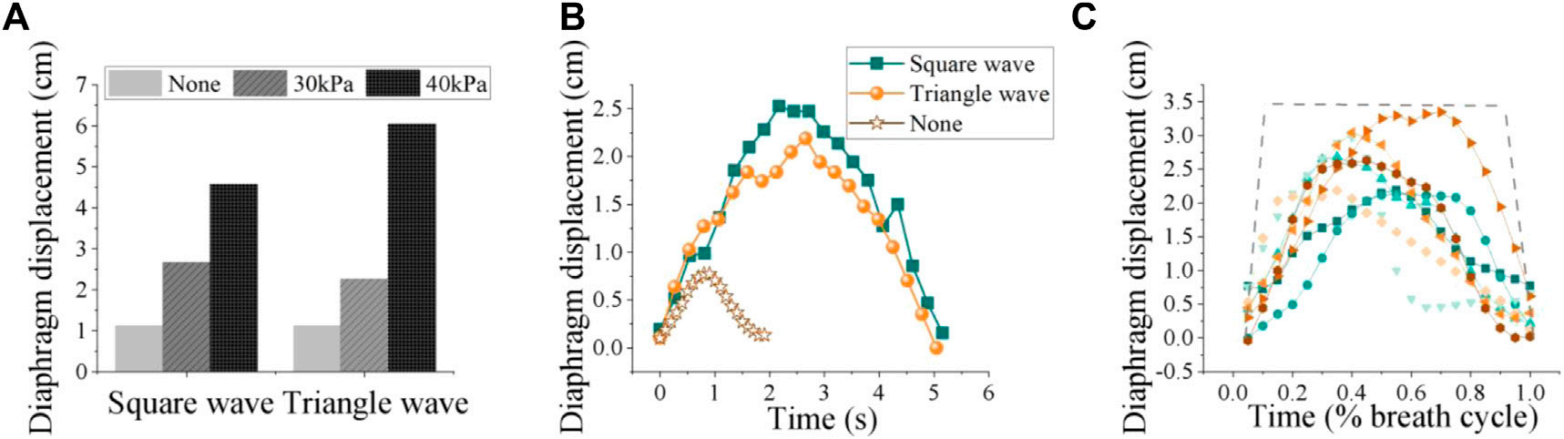
**(A)** Diaphragm displacement: input waveforms, including square and triangle shapes, at both 0 kPa, 30 kPa and 40 kPa pressure levels. **(B)** Effects of different pneumatic waveforms on diaphragm curve (30 kPa). **(C)** Diaphragm motion patterns under the same robotic forces varied widely among participants and spanned a large range. The lines represent measured diaphragm motion via ultrasound after image processing, normalized to the breath cycle.

As depicted in Figure 11A, the increase in robotic pressure significantly enhanced diaphragm displacement for both square and triangle waveforms compared to no assistance. To further explore the influence of different robotic pressurization patterns on diaphragm motion, we employed image segmentation to capture the diaphragm curve in ultrasonography (Mukhopadhyay and Chaudhuri, 2015). The results indicate that the various waveforms do not significantly affect the diaphragm displacement curve, unlike the amplitude (Figure 11B).

Therefore, the basic pattern of diaphragmatic motion tends to relatively consistent regardless of the specific waveform of assistance, but the pressure amplitude might affect how forcefully the diaphragm contracts and, consequently, the peak displacement it achieves during each breath. Thus, in the design of robotic systems, the magnitude of the pressure is paramount; the configuration of the waveform can incorporate a selection of common settings.

Diaphragm motion curves for eight subjects under the same robotic assistance force are shown in Figure 11C. We calculated the average diaphragm motion curve for each participant over three consecutive breathing cycles, selecting 20 data points from each cycle for analysis. The results revealed significant individual variability in diaphragm motion patterns, even when the same robotic assistance was applied. This variability likely arises from complex interactions within the musculoskeletal and nervous systems. The variation in diaphragm movement among individuals under the same robotic assistance conditions may point to a significant finding from our previous research: the effects of robotic respiratory support vary significantly among different individuals. Therefore, the development of personalized assistance based on muscle force has the potential to address these individual differences during robotic assistance, paving the way for more customized and effective solutions. These findings could impact clinical practice by favoring patient comfort and ease in designing respiratory assistive devices.

## 5 Discussion

The diaphragm is the primary respiratory muscle, making it essential to assess its function in response to respiratory assist devices to avoid injury and enhance the effectiveness and comfort of the assistance provided. However, existing devices that apply pressure to the abdominal cavity can aid weakened respiration, but this approach shows limitations when assessed from the perspective of diaphragmatic motion. Therefore, in this study, we utilize diaphragm ultrasound to evaluate the effect of soft robotic ventilation and propose a human-robot coupled two-compartment respiratory mechanic model to quantify the robotic assistance on diaphragm displacement and lung ventilation.

The primary findings of this article strongly suggest that the extracorporeal respiratory intervention mechanism, which applies pressure to the abdominal muscles to push the diaphragm upward and thereby increase ventilation, is feasible. This non-invasive respiratory assistance not only effectively enhances ventilation but also prevents disuse atrophy of the diaphragm. Moreover, this study not only demonstrates the rationality and potential of proposed robotic assistive ventilation but also highlights its superiority in preventing diaphragmatic injury compared to clinical positive pressure ventilation.

Here, we elaborate on three significant aspects of our findings through comparative analysis with other research. First, we utilized ultrasound imaging in humans for the first time to examine the diaphragm’s “under the skin” movements during “out of skin” robotic interventions. By injecting respiratory mechanics into diaphragm ultrasound evaluation, we expect the biomimetic diaphragm ultrasound might provide a better understanding of the effect of robotic assistance on human-robot respiratory system.

Second, although diaphragm ultrasound provides vital information about health and diseases by capturing signals and dynamics of internal organs, evaluating diaphragm functions and diagnosing conditions based on this method is not straightforward for clinicians. Currently, specific ranges of diaphragm ultrasound indicators are derived from extensive experimental statistics. However, the considerable variability and wide range of clinical data make it challenging to rely solely on these indicators for clinical diagnoses. The development of a human-robot integrated two-compartment mechanical model offers clinicians valuable insights into the dynamics of diaphragm-thoracoabdominal-lung system when assisted by robotics. This advancement enables clinicians to achieve a more intuitive understanding of the respiratory process, thereby enabling precise diagnoses and the customization of personalized robotic parameters for respiratory rehabilitation.

Third, consistent with the research by Lucy Hu and Lee, synchronization between the human and the machine during respiratory assistance is extremely important. Both studies trigger the robot through airflow, thereby achieving synchronization between the robot’s assistive force and the individual’s own efforts. Our observations via diaphragmatic ultrasonography reveal significant variability in the timing from the onset of diaphragmatic contraction to its maximum during respiration among individuals. Optimizing the robot’s assistive force to align with the timing of the diaphragm’s initial contraction could further enhance human-machine synchronization.

Finally, even though, on an aggregate level, the soft robotic system tends to improve ventilation, the individual responses differ. These findings underscore the significance of acknowledging individual variability (Malcolm, 2013; Zhang et al., 2017) and the intricate dynamics of human-robot interaction in the context of respiratory assistance. Future research endeavors may be poised to explore the development of tailored respiratory support systems, including the development of controllers explicitly derived from biological mechanisms that capture the diaphragm muscle dynamics and the implementation of a human-in-the-loop optimization method for individualized robotic assistance.

In future work, we will conduct studies on the optimization design and personalized assistance of ultrasound-guided wearable soft robotic respiratory assistive exoskeletons. Specifically, using multi-modal signals allows for a more precise assessment of the effectiveness of robot-assisted interventions; thus, combining diaphragm ultrasound with other respiratory indicators, such as chest movement, airflow, blood oxygen saturation, and other clinical monitoring parameters, can help determine the appropriate level of robot assistance. Additionally, in this study, ultrasound was solely used for monitoring purposes; however, in the future, real-time ultrasound imaging signals will be incorporated into the design of assistive robotic devices with closed-loop control. Moreover, the current experimental study only included healthy participants; nevertheless, future research will validate the effectiveness of robots in patients with respiratory disorders, especially those with diaphragm paralysis.

## 6 Conclusion

In this paper we propose a noninvasive, *in vitro*, clinically easy-to-carry-out ultrasound method to evaluate the effect of soft robotic assistive ventilation. Diaphragm displacement during robotic ventilation is chosen to gauge the impact of robotic assistive forces on the human respiratory system. The relationship between diaphragm displacement under different robotic assistive patterns and ventilation parameters is studied across eight healthy participants. To elucidate the underlying pressure transmission mechanism with robotic assistance, we establish the human-robot coupled two-compartment respiratory mechanic model and make a comparison with experimental results.

Compared with invasive ventilator devices, the soft robot in this study can reduce lung injury and enhance comfort by assisting respiration through emulation of natural human breath. Compared with other non-invasive respiratory assistive devices, the soft robot in this study adopts soft silicone as the production material, which is comfortable to wear and non-destructively to biological tissue.; Besides, the origami-inspired design ensures the lightweight in the resting state and the large volume variation in the working state. Finally, this soft robot is cost-effective, making it suitable for widespread clinical and home-use.

Future work would need to be performed to evaluate how the biomimetic respiratory evaluation might be used for optimizing and guiding robotic design and individualized controller design for variable respiratory dysfunctions.

## Data Availability

The raw data supporting the conclusions of this article will be made available by the authors, without undue reservation.
